# Tyrosine Kinase Inhibitor Therapy in Metastatic Medullary Thyroid Carcinoma: Real-World Data from Turkish Oncology Group

**DOI:** 10.3390/jcm15062353

**Published:** 2026-03-19

**Authors:** Sedat Yıldız, Hacer Demir, Talha Özüdoğru, Damla Günenç, Zeynep Sıla Gökdere, Hayati Arvas, Zuhat Urakçı, Seda Jeral Evinç, Özkan Alan, Rumeysa Çolak, Mesut Yılmaz, Esra Aşık, Atila Yıldırım, Ali Kaan Güren, Osman Köstek, Berkan Karabuğa, Öztürk Ateş, Canberk Şencan, Tuğba Yavuzşen, Şuheda Ataş İpek, İsmail Oğuz Kara, Teoman Şakalar, Ahmet Cebeli Gökay, Havva Yeşil Çınkır, Ahmet Kürşad Dişli, Mevlüde İnanç, Olçun Ümit Ünal, Emre Yılmaz, İlhan Hacıbekiroğlu, Sait Kitaplı, Özgür Tanrıverdi, Elif Şahin, Muhammed Fatih Sağıroğlu, Pembegül Yumuştutan, Seray Saray, Selahattin Çelik, Hayriye Şahinli, Azer Gökmen, Gizem Bakır Kahveci, Didem Divriklioğlu, Saadettin Kılıçkap

**Affiliations:** 1 Department of Medical Oncology, Afyonkarahisar Health Sciences University, Afyonkarahisar 03030, Turkey; drhacerdemir@gmail.com; 2 Department of Medical Oncology, Faculty of Medicine, Ege University, İzmir 35100, Turkey; talhaozudogru@hotmail.com (T.Ö.); d.gunenc@yahoo.com.tr (D.G.); silagokdere@hotmail.com (Z.S.G.); 3 Department of Medical Oncology, Faculty of Medicine, Dicle University, Diyarbakır 21280, Turkey; hayatiarvas65@gmail.com (H.A.); dr.zurak@hotmail.com (Z.U.); 4 Department of Medical Oncology, Cerrahpaşa Faculty of Medicine, Istanbul University, Istanbul 34452, Turkey; sedajeral@gmail.com (S.J.E.); ozkan.alan@hotmail.com (Ö.A.); 5 Department of Medical Oncology, Bakırköy Dr. Sadi Konuk Training and Research Hospital, Istanbul 34147, Turkey; colak.rmys@gmail.com (R.Ç.); mesutyilmaz12@yahoo.com (M.Y.); 6 Department of Medical Oncology, Faculty of Medicine, Karadeniz Technical University, Trabzon 61080, Turkey; dr.esrakaya@hotmail.com (E.A.); dr_atila_yildirim@hotmail.com (A.Y.); 7 Department of Medical Oncology, Faculty of Medicine, Marmara University, Istanbul 34854, Turkey; alikaanguren@gmail.com (A.K.G.); osmankostek@hotmail.com (O.K.); 8 Department of Medical Oncology, Dr. Abdurrahman Yurtaslan Ankara Oncology Training and Research Hospital, Ankara 06200, Turkey; drbkarabuga@gmail.com (B.K.); dr.ozturkates@gmail.com (Ö.A.); 9 Department of Medical Oncology, Faculty of Medicine, Dokuz Eylül University, İzmir 35330, Turkey; canberksencan3@gmail.com (C.Ş.); drtugba@yahoo.com (T.Y.); 10 Department of Medical Oncology, Faculty of Medicine, Çukurova University, Adana 01790, Turkey; suhedaatas92@gmail.com (Ş.A.İ.); iokaracu@cu.edu.tr (İ.O.K.); 11 Department of Medical Oncology, Faculty of Medicine, Kahramanmaraş Sütçü İmam University, Kahramanmaraş 46050, Turkey; drteomansakalar@gmail.com; 12 Department of Medical Oncology, Faculty of Medicine, Gaziantep University, Gaziantep 27850, Turkey; drcebeli@gmail.com (A.C.G.); drhavva1982@gmail.com (H.Y.Ç.); 13 Department of Medical Oncology, Erciyes University, Kayseri 38280, Turkey; kdisli@gmail.com (A.K.D.); mevludeinanc@hotmail.com (M.İ.); 14 Department of Medical Oncology, İzmir City Hospital, İzmir 35540, Turkey; drolcun@gmail.com; 15 Department of Medical Oncology, Faculty of Medicine, Sakarya University, Sakarya 54290, Turkey; emreyilmz@gmail.com (E.Y.); ilhanhbo@hotmail.com (İ.H.); 16 Department of Medical Oncology, Faculty of Medicine, Muğla Sıtkı Koçman University, Muğla 48000, Turkey; kitaplisait@gmail.com (S.K.); drozgurt@gmail.com (Ö.T.); 17 Department of Medical Oncology, Kocaeli City Hospital, Kocaeli 41060, Turkey; 18 Department of Medical Oncology, Antalya City Hospital, Antalya 07080, Turkey; dr.mfsagiroglu@gmail.com; 19 Department of Medical Oncology, Ümraniye Training and Research Hospital, Istanbul 34764, Turkey; yumustutan@gmail.com; 20 Department of Medical Oncology, Balıkesir Atatürk City Hospital, Balıkesir 10100, Turkey; drseraysaray@gmail.com; 21 Department of Medical Oncology, Etlik City Hospital, Ankara 06170, Turkey; drcelikselahattin@gmail.com (S.Ç.); dr.hayriye@hotmail.com (H.Ş.); 22 Department of Medical Oncology, Van Yüzüncü Yıl University, Van 65090, Turkey; azergkmen90@gmail.com; 23 Department of Medical Oncology, Faculty of Medicine, Trakya University, Edirne 22030, Turkey; bakirkahvecigizem@gmail.com (G.B.K.); dr_didemeroglu@hotmail.com (D.D.); 24 Department of Medical Oncology, LIV Hospital Ankara, Ankara 06680, Turkey; skilickap@yahoo.com

**Keywords:** medullary thyroid carcinoma, tyrosine kinase inhibitor

## Abstract

**Background:** Vandetanib and cabozantinib are the approved first-line antiangiogenic multikinase inhibitors (aaMKIs) for metastatic medullary thyroid carcinoma (MTC); however, real-world data on their comparative efficacy, optimal sequencing, and outcomes beyond the first-line setting remain limited. We report multicenter real-world outcomes from a large Turkish cohort. **Methods:** In this retrospective multicenter cohort study, we analyzed data from 24 oncology referral centers across Türkiye. Patients with histologically confirmed metastatic MTC who received systemic therapy between December 2011 and December 2024 were included. The primary endpoint was progression-free survival (PFS), assessed separately for first-line (PFS1) and second-line (PFS2) therapy. Overall survival (OS) and prognostic factors were evaluated using Kaplan–Meier and Cox proportional hazards analyses. **Results:** A total of 115 patients were included (median age 47.4 years; 63.5% male). In the first-line setting, vandetanib (47.8%) and cabozantinib (30.4%) were the most frequently used agents. Median PFS1 was 40.8 months with vandetanib and was not reached with cabozantinib; both were significantly superior to chemotherapy (median PFS1 4.9 months; log-rank *p* < 0.001). In the second-line setting, median PFS2 was not reached with cabozantinib and was 32.5 months with vandetanib. Sequential use of cabozantinib and vandetanib across the first two lines was associated with a median time to second progression of 114 months, compared with 39 months in patients receiving any other TKI combination (*p* = 0.003). Second-line use of cabozantinib or vandetanib was independently associated with improved OS (HR 0.40, 95% CI 0.16–0.98; *p* = 0.046). On multivariate analysis, younger age (HR 0.16, 95% CI 0.03–0.72; *p* = 0.017) and bone metastasis (HR 0.29, 95% CI 0.11–0.73; *p* = 0.009) were independent prognostic factors for OS. **Conclusions:** In this real-world cohort of patients with metastatic MTC, cabozantinib and vandetanib demonstrated durable efficacy across treatment lines, substantially outperforming alternative TKIs and chemotherapy. Sequential use of both approved aaMKIs was associated with prolonged disease control. These findings suggest a potential association between access to both agents and improved outcomes. They are consistent with their central role in treatment sequencing, particularly in settings with limited access to selective RET inhibitors. Given the retrospective design and small subgroup sizes, these results should be interpreted as exploratory and hypothesis-generating.

## 1. Background

Medullary thyroid carcinoma (MTC) is a rare neuroendocrine malignancy arising from the parafollicular C cells of the thyroid gland, which secrete calcitonin [1]. Accounting for 5–10% of all thyroid carcinomas and approximately 0.4–1.4% of thyroid nodules, MTC occupies a distinct clinical and biological niche within the spectrum of thyroid malignancies [2]. The disease is classified into sporadic and hereditary forms; the latter, comprising approximately 25% of cases, occurs in the context of Multiple Endocrine Neoplasia type 2A (MEN2A) or 2B (MEN2B) syndromes, or as familial MTC, and is caused by germline pathogenic variants in the RET proto-oncogene [3]. Sporadic MTC, which constitutes the majority of cases, is frequently associated with somatic RET mutations—most commonly the M918T variant—as well as RAS mutations in a subset of RET-wild-type tumors [4].

While localized MTC is potentially curable with total thyroidectomy and central lymph node dissection, outcomes for patients with metastatic disease are considerably worse. The 10-year survival rate for patients presenting with distant metastases at diagnosis has been reported at approximately 20%, underscoring the urgent need for effective systemic therapies [3]. Distant metastases most frequently involve the liver, lung, bone, and distant lymph nodes, and the pattern of organ involvement carries prognostic significance, with hepatic and bone metastases generally associated with shorter survival [5]. Elevated calcitonin and carcinoembryonic antigen (CEA) doubling times have long served as dynamic biomarkers of disease progression, with shorter doubling times portending a more aggressive clinical course [6].

The identification of RET and RAS mutations as central oncogenic drivers in MTC has fundamentally shaped the therapeutic landscape. The RET proto-oncogene encodes a receptor tyrosine kinase involved in cell proliferation, survival, and differentiation; its constitutive activation through mutation leads to uncontrolled tumor growth. This biologic insight paved the way for targeted inhibition as a therapeutic strategy, initially through multitargeted antiangiogenic kinase inhibitors and subsequently through highly selective RET-directed agents [7].

Vandetanib and cabozantinib, both orally administered antiangiogenic multikinase inhibitors (aaMKIs) with non-selective RET inhibitory activity, represent the established backbone of systemic therapy for metastatic MTC in patients without actionable RET mutations or in settings where selective RET inhibitors are unavailable. Vandetanib, approved by the U.S. Food and Drug Administration in 2011, targets VEGFR, RET, and EGFR, and demonstrated a significant progression-free survival (PFS) benefit over placebo in a pivotal phase III trial, with an estimated median PFS of 30.5 months in the treatment arm [8]. Cabozantinib, approved in 2012, inhibits VEGFR1/2, c-MET, and RET and showed a median PFS of 11.2 months versus 4.0 months with placebo in the EXAM trial, with a clinically meaningful, though statistically non-significant, trend toward improved overall survival [9]. Both agents carry a substantial toxicity burden, including hypertension, diarrhea, palmar-plantar erythrodysesthesia, QTc prolongation, and hepatotoxicity, which frequently require dose modification and multidisciplinary supportive management [10].

More recently, the development of highly selective RET kinase inhibitors—selpercatinib and pralsetinib—has further refined the treatment algorithm for RET-mutant MTC. In the phase III LIBRETTO-531 trial, selpercatinib demonstrated superior PFS compared with vandetanib or cabozantinib in patients with RET-mutant advanced MTC, establishing its preferred status in this molecular subgroup [11]. Despite these advances, access to molecular testing and novel targeted agents remains inconsistent globally, particularly in lower-resource healthcare systems, where vandetanib and cabozantinib remain the primary treatment options for most patients.

For patients progressing on first-line aaMKI therapy, current guidelines endorse the sequential use of an alternative approved aaMKI or treatment with sorafenib, sunitinib, or Lenvatinib. However, the level of evidence supporting these options is considerably weaker and derived largely from small retrospective series and case reports [12]. The optimal sequencing of available agents—particularly the relative merits of deploying cabozantinib versus vandetanib as initial versus subsequent therapy—has not been prospectively evaluated, and real-world outcome data to guide these decisions are scarce.

The present study was designed to address this evidence gap. Using a large retrospective multicenter cohort assembled across 24 oncology referral centers in Türkiye, we sought to evaluate the comparative efficacy of TKIs across treatment lines in metastatic MTC, characterize the impact of treatment sequencing on disease control and survival, and identify clinical and molecular factors independently associated with patient outcomes in a real-world setting.

## 2. Methods

### 2.1. Study Design and Patient Population

This retrospective, multicenter study analyzed data from 24 oncology referral centers across different regions of Türkiye. Patients with histologically confirmed medullary thyroid carcinoma (MTC) diagnosed between December 2011 and December 2024 were identified through institutional databases.

A total of 121 patients were initially screened. Patients with a history of secondary malignancies, those with poor functional status (Eastern Cooperative Oncology Group [ECOG] performance status 3–4), or those who had not received any form of systemic anticancer therapy were excluded. After applying these exclusion criteria, 115 patients with metastatic disease—either at initial diagnosis or during follow-up—were included in the final analysis.

### 2.2. Treatment and Data Collection

Clinical, demographic, and pathological data were retrospectively collected from medical records. Information regarding systemic treatments, including multikinase tyrosine kinase inhibitors, chemotherapy, and selective RET inhibitors, was recorded in detail, along with treatment sequencing and line of therapy.

Radiological evaluations were performed according to institutional standards. Tumor response and disease progression were assessed according to the Response Evaluation Criteria in Solid Tumors (RECIST) version 1.1.

### 2.3. Outcomes

The primary endpoint of the study was progression-free survival (PFS), defined as the time from initiation of each systemic treatment to the first documented radiological progression or death from any cause. PFS was evaluated separately for first-line (PFS1) and subsequent lines of therapy (PFS2).

### 2.4. Statistical Analysis

Descriptive statistics were used to summarize baseline demographic and clinicopathological characteristics. Progression-free survival was estimated using the Kaplan–Meier method and compared between treatment groups using the log-rank test. Cox proportional hazards regression models were applied to estimate hazard ratios (HRs) and corresponding 95% confidence intervals (CIs). For multivariable Cox regression analyses, variables were selected based on *p* values < 0.05 in univariate analyses, in addition to clinically relevant covariates. The proportional hazards assumption was evaluated using graphical inspection of log-minus-log survival plots. Missing data were handled using complete-case analysis, given the retrospective nature of the dataset. A two-sided *p* value of less than 0.05 was considered statistically significant. All statistical analyses were conducted using SPSS software version 27 (IBM Corp., Armonk, NY, USA).

### 2.5. Ethics Statement

The study was conducted in accordance with the Declaration of Helsinki. Ethical approval was obtained from the local institutional review boards of the participating centers. Given the retrospective nature of the study, informed consent was waived (Approval Code: 2025/15; Approval Date: 5 December 2025).

## 3. Results

### Patient Demographics and Baseline Characteristics

A total of 115 patients with metastatic medullary thyroid carcinoma were included in the final analysis. Baseline demographic and clinicopathological characteristics are presented in Table 1. The median age at diagnosis was 47.4 years (range, 11–88), and 63.5% of the cohort were male. At the initiation of systemic therapy, the majority of patients had an Eastern Cooperative Oncology Group (ECOG) performance status of 0–1.

Metastatic disease was present at initial diagnosis in 47.8% of patients, whereas 52.2% developed distant metastases during follow-up. The most frequent metastatic sites were the lung, liver, bone, and distant lymph nodes, with a substantial proportion of patients exhibiting involvement of multiple organ systems. The RET mutation status was available in 70 patients, of whom 20.9% harbored activating RET mutations.

The distribution of key baseline characteristics across first-line treatment groups is summarized in Table 1. The majority of patients in all treatment groups had an ECOG performance status of 0–1 at treatment initiation (95.7% overall). Regarding metastatic sites, lymph node involvement was the most frequent finding across groups, followed by lung, bone, and liver metastases. RET mutation testing was performed in varying proportions across treatment subgroups. Known RET mutation positivity rates were 14.8% in the vandetanib group, 35.3% in the cabozantinib group, 23.1% in the chemotherapy group, and 0% in the sorafenib group. These differences in RET mutation rates across treatment groups likely reflect treatment allocation practices, as patients with known RET mutations may have been preferentially directed toward specific agents or clinical trials. Demographic characteristics, including age and sex distribution, were broadly comparable across groups. Due to the retrospective design and the relatively small size of individual treatment subgroups, formal statistical comparisons of baseline characteristics across groups were not performed; potential confounding by baseline differences should therefore be considered when interpreting the comparative efficacy results.

**Figure 1:**
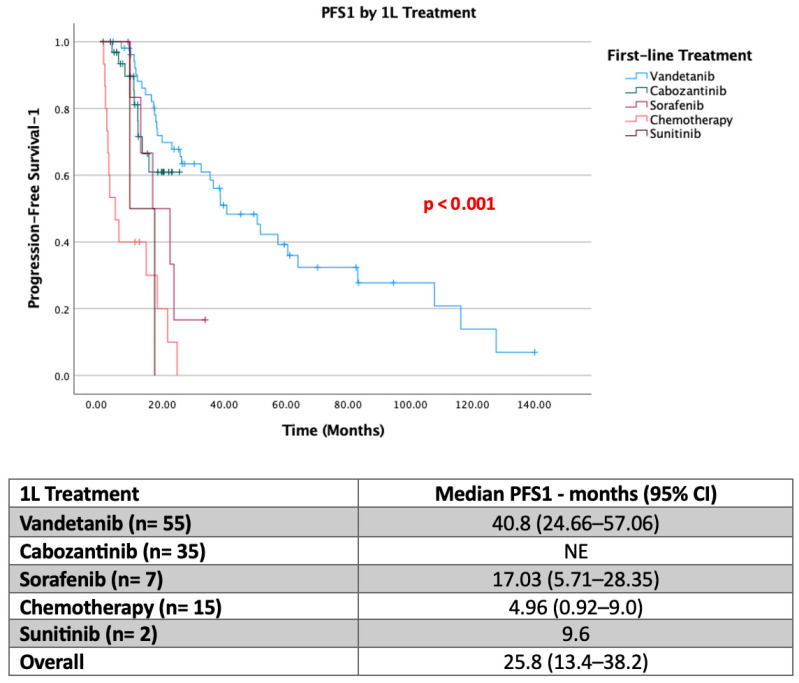
PFS data of first-line treatments in metastatic thyroid medullary carcinoma (PFS1). NE, Not Estimable (median could not be calculated due to insufficient events at the time of analysis).

In first-line treatment, 47.8% (*n* = 55) of patients received Vandetanib, 30.4% (*n* = 35) received Cabozantinib, and 13% (*n* = 15) received chemotherapy. Median PFS1 was not reached in the Cabozantinib group. Sixty percent of patients continued treatment during the 25-month treatment period. In the Vandetanib group, the median PFS1 was 40.8 months. In the Sorafenib arm, the median PFS1 was 17 months; in the Sunitinib arm, 9.6 months; and in the chemotherapy arm, 4.9 months (Figure 1). Specifically, in first-line treatment, 10.4% (*n* = 12) (11 in the Vandetanib group) experienced treatment termination due to drug supply problems.

Vandetanib achieved a median first-line progression-free survival (PFS1) of 40.8 months. In patients treated with cabozantinib as first-line therapy, the median PFS1 was not reached at the time of data cutoff, indicating sustained disease control in a substantial proportion of patients. Sorafenib demonstrated a median PFS1 of 17.0 months, while patients receiving chemotherapy experienced markedly inferior outcomes.

Kaplan–Meier analysis revealed a statistically significant difference in PFS1 across treatment groups (log-rank *p* < 0.001; Figure 1). Compared with chemotherapy, treatment with vandetanib and cabozantinib was associated with a significantly prolonged PFS1. Sorafenib conferred intermediate efficacy, with outcomes superior to chemotherapy but inferior to those of approved multikinase inhibitors.

In second-line therapy, 36.6% (*n* = 26) of patients received cabozantinib, 21.1% (*n* = 15) received vandetanib, and 21.1% (*n* = 15) received sorafenib. Similar to PFS1, the median PFS2 was not reached in the cabozantinib group, and more than 60% of patients continued treatment at 80 months. In the vandetanib group, the median PFS2 was 32.5 months. In the sorafenib arm, the median PFS2 was 17.8 months; in the sunitinib arm, 6.2 months; in the lenvatinib arm, 6.1 months; and in the chemotherapy arm, only 4.3 months.

In the second-line treatment setting, cabozantinib continued to demonstrate durable efficacy, with median PFS2 not reached at the time of analysis. Vandetanib and sorafenib were associated with median PFS2 values of 32.5 months and 17.8 months, respectively. Chemotherapy remained associated with the shortest progression-free survival.

**Figure 2:**
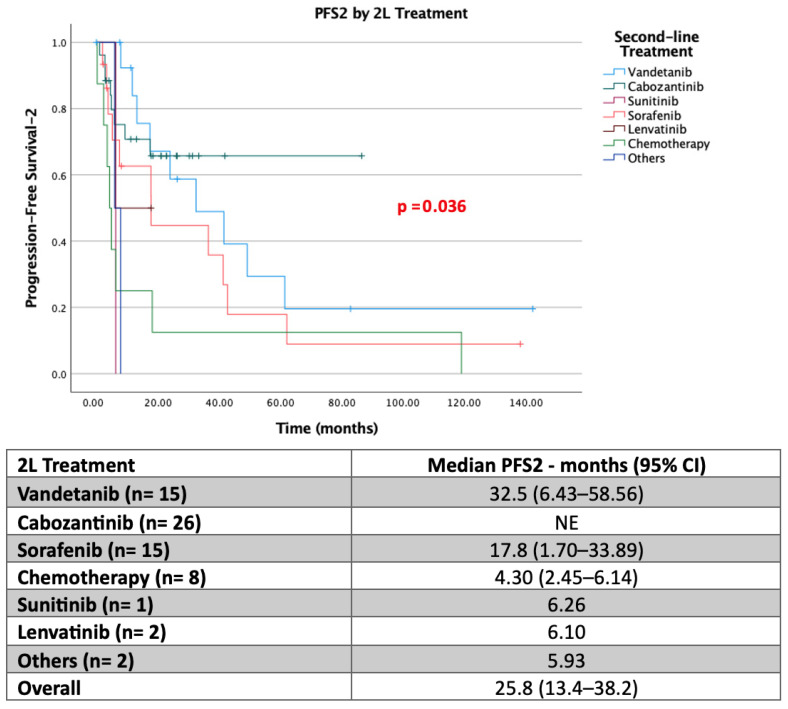
PFS data of second-line treatments in metastatic thyroid medullary carcinoma (PFS2). NE, Not Estimable (median could not be calculated due to insufficient events at the time of analysis).

Kaplan–Meier estimates showed significant separation of survival curves among second-line treatment groups (log-rank *p* < 0.001; Figure 2), confirming the sustained benefit of tyrosine kinase inhibitors beyond the first-line setting.

**Figure 3:**
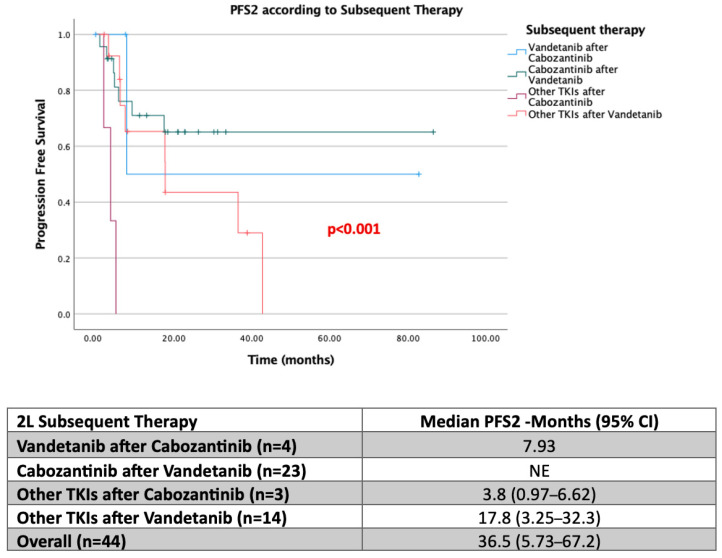
Second-line progression-free survival (PFS) outcomes according to first-line treatment selection. NE, Not Estimable (median could not be calculated due to insufficient events at the time of analysis).

When the efficacy of second-line tyrosine kinase inhibitors (TKIs) following first-line Cabozantinib or Vandetanib use was evaluated, the median PFS2 was not reached in patients who received Cabozantinib as second-line therapy after first-line Vandetanib. In contrast, the median PFS2 was calculated as 17.7 months for other TKIs administered after Vandetanib. Although the dataset was limited for evaluating the efficacy of Vandetanib or other TKIs following Cabozantinib, the median PFS2 was 7.93 months in patients who received Vandetanib after Cabozantinib, whereas it was 3.8 months for other TKIs used after Cabozantinib (Figure 3).

**Figure 4:**
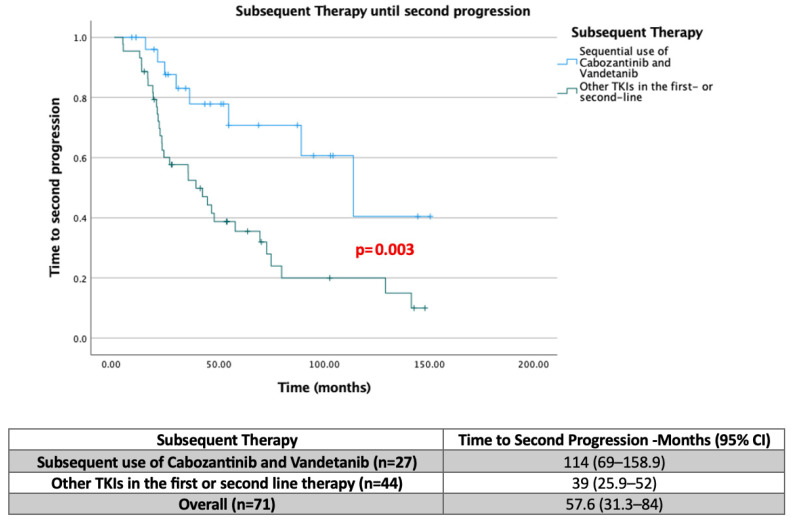
Subsequent use of Cabozantinib and Vandetanib vs. other TKIs.

The most striking difference emerged with the sequential use of cabozantinib and vandetanib. When the time to second progression was evaluated, the median time was 114 months in patients who received both cabozantinib and vandetanib across the first two lines of therapy, compared with 39 months in patients who received any other TKI in either the first or second line (Figure 4). This difference was statistically significant (*p* = 0.003).

When overall survival data were assessed, no significant individual survival benefit was observed for any single TKI. Notably, chemotherapy performed worse than all TKI regimens across the cohort.

**Figure 5:**
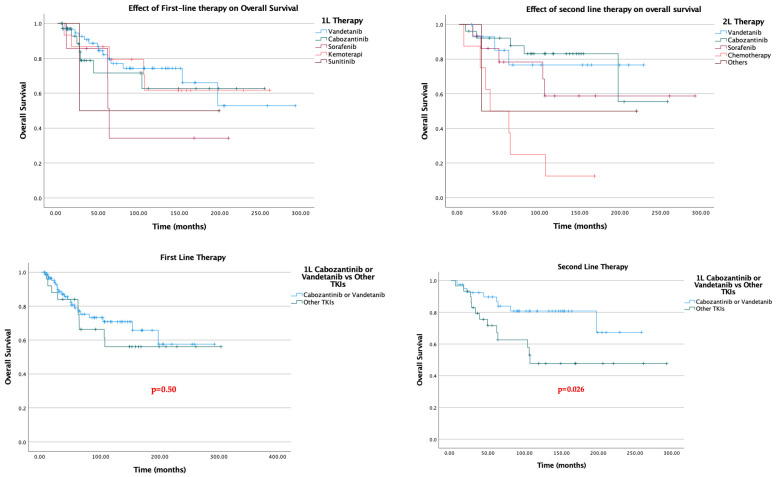
Effects of the First- and Second-line use of TKIs on overall survival.

In contrast, when compared with other TKIs, first-line use of cabozantinib or vandetanib did not confer a significant overall survival advantage. However, the critical determinant was the selection of second-line treatment. Regardless of which TKI was used in the first line, the use of cabozantinib or vandetanib in the second line was associated with a significant overall survival benefit (*p* = 0.026) (Figure 5).

**Figure 6:**
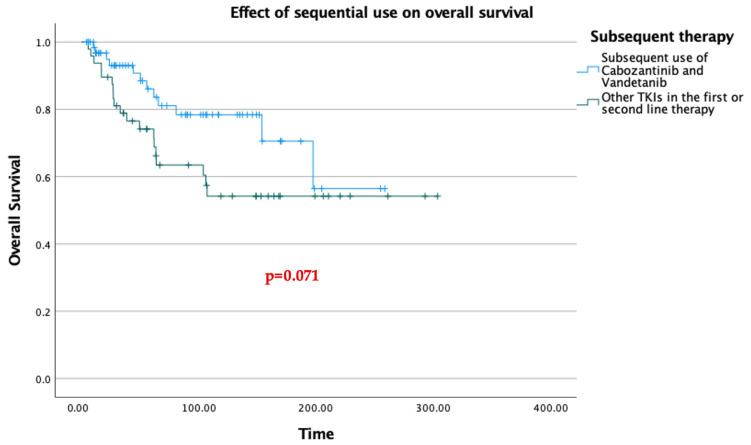
Effect of sequential use of Cabozantinib and Vandetanib vs. other TKIs.

The survival advantage observed with sequential cabozantinib and vandetanib in PFS analysis was not mirrored in overall survival (Figure 6). Median overall survival was not reached in either group, and no statistically significant difference was observed (*p* = 0.071). Nevertheless, the key clinical implication of this finding is that exposure to either cabozantinib or vandetanib at any point during the treatment course—even without strict sequential use across the first two lines—appeared to contribute meaningfully to overall survival.

Overall survival (OS) was evaluated using univariate and multivariate Cox regression analyses (Table 2). In the univariate analysis, age < 40 years was significantly associated with improved OS compared to ≥40 years (HR: 0.19, 95% CI: 0.06–0.65, *p* = 0.008). This association remained statistically significant in the multivariate model (HR: 0.16, 95% CI: 0.03–0.719, *p* = 0.017), indicating that younger age was an independent favorable prognostic factor.

Gender was also significantly associated with OS in the univariate analysis, with females demonstrating better survival compared to males (HR: 0.40, 95% CI: 0.16–0.98, *p* = 0.047). However, this association lost statistical significance in the multivariate analysis (HR: 0.42, 95% CI: 0.12–1.46, *p* = 0.174), suggesting that the observed effect may be confounded.

Among metastatic sites, the presence of bone metastasis was significantly associated with improved OS in the univariate analysis (HR: 0.48, 95% CI: 0.23–0.98, *p* = 0.046) and remained an independent prognostic factor in the multivariate model (HR: 0.29, 95% CI: 0.11–0.73, *p* = 0.009). This finding indicates that patients with bone metastasis had a lower hazard of death compared to those without bone metastasis in this cohort. In contrast, lymph node, lung, and liver metastases were not significantly associated with OS.

With respect to treatment-related variables, the use of Cabozantinib or Vandetanib in the first-line setting was not significantly associated with OS. However, second-line use of Cabozantinib or Vandetanib was significantly associated with improved survival in both univariate (HR: 0.38, 95% CI: 0.15–0.92, *p* = 0.032) and multivariate analyses (HR: 0.40, 95% CI: 0.163–0.983, *p* = 0.046), suggesting that subsequent therapy with these tyrosine kinase inhibitors may confer a survival benefit.

Other evaluated variables, including family history, smoking status, alcohol use, comorbidity, calcitonin level, CEA level, RET mutation status, and other metastatic sites, were not significantly associated with overall survival in this cohort.

Overall, younger age, presence of bone metastasis, and second-line Cabozantinib or Vandetanib use emerged as significant prognostic factors for overall survival in this study population.

## 4. Discussion

Pathogenic variants in the RET proto-oncogene can be detected in the majority of medullary thyroid cancer (MTC) cases. Current guidelines recommend a selective RET kinase inhibitor (selpercatinib) rather than antiangiogenic multikinase inhibitors (aaMKIs) for patients harboring somatic or germline RET mutations, based on improved outcomes observed in open-label, randomized, and non-randomized studies, as well as a relatively more favorable toxicity profile compared with aaMKIs [9,11].

The nature of the underlying RET mutation may influence treatment outcomes. Unlike vandetanib or cabozantinib, selpercatinib appears to have strong inhibitory activity against the “gatekeeper” mutation at RET codon 804 [12]. However, mutations at codon 810 may confer resistance to selpercatinib, and such mutations have been reported in patients treated with selective RET inhibitors [12]. In our study, due to the limited number of patients receiving selpercatinib, a robust efficacy evaluation could not be performed.

In developing countries, treatment strategies are often shaped by national conditions and drug accessibility. According to current guidelines, vandetanib or cabozantinib is recommended as a first-line option for patients without targetable RET (germline or somatic) mutations or for those who are unwilling or unable to participate in clinical trials. For patients who progress on one or both of these agents, sorafenib, sunitinib, or lenvatinib is a reasonable alternative.

In randomized trials evaluating aaMKIs with non-selective RET-inhibitory activity (e.g., vandetanib and cabozantinib), partial response rates have ranged from approximately 20% to 60% [10,13].

Vandetanib is an oral inhibitor targeting VEGFR, RET, and epidermal growth factor receptor (EGFR) [14]. It was initially evaluated in a phase II trial involving 30 patients with metastatic or unresectable hereditary MTC (300 mg daily) [15]. Subsequently, an international randomized phase III trial including more than 300 patients with locally advanced or metastatic sporadic or hereditary unresectable MTC demonstrated that vandetanib significantly prolonged progression-free survival (PFS) compared with placebo (HR 0.46, 95% CI 0.31–0.69, *p* < 0.001) [16]. The median PFS had not yet been reached in the vandetanib arm at the time of analysis, but was estimated at 30.5 months compared with 19.3 months in the placebo group. The objective response rate was significantly higher in the vandetanib group (45% vs. 13%). Consistent with the literature, our study demonstrated a median PFS of 40.8 months in the first-line vandetanib group.

Cabozantinib is an oral small-molecule kinase inhibitor targeting VEGFR 1 and 2, c-MET, and RET [17]. Its inhibitory activity against c-MET, the cognate receptor of hepatocyte growth factor, may provide additional synergistic benefit in MTC [17]. In a phase I study, confirmed partial responses were observed in 29% of patients with MTC [18]. A randomized trial involving 330 patients with metastatic or unresectable locally advanced MTC demonstrated a significant improvement in PFS with cabozantinib compared to placebo (11.2 vs. 4.0 months; HR 0.28, 95% CI 0.19–0.40) [19]. Partial responses were observed in 27% of patients receiving cabozantinib and in none of those receiving placebo. Although not statistically significant, a clinically meaningful improvement in overall survival (OS) of 5.5 months was noted in favor of cabozantinib (26.6 vs. 21.1 months).

Subgroup analyses revealed that patients harboring RET M918T or RAS mutations derived substantial PFS benefit from cabozantinib compared to placebo. Moreover, in a post hoc analysis, patients with RET M918T mutations demonstrated a significant OS improvement (44.3 vs. 18.9 months; HR 0.60, 95% CI 0.38–0.94) [20].

In our study, median PFS was not reached with cabozantinib in either the first- or second-line setting. In the third-line setting, although the patient number was limited (*n* = 9), cabozantinib demonstrated a median PFS of 36.9 months, indicating notable activity even in later treatment lines.

An important observation concerned patients progressing on vandetanib. In those who received vandetanib in the first line, median PFS2 was not reached with second-line cabozantinib, whereas it was 17.7 months with other TKIs. This finding suggests that cabozantinib remains highly effective after vandetanib failure. Conversely, after first-line cabozantinib, median PFS2 was 7.9 months with vandetanib and only 3.8 months with other TKIs, indicating shorter response durations with alternative agents following cabozantinib.

RET mutation status remains a critical parameter in treatment planning for metastatic MTC. In our study, RET mutation analysis was performed in 63.6% of patients, and 21.8% were found to harbor a RET mutation. In the first-line vandetanib group, median PFS1 was 38.8 months in RET-negative patients and 40.8 months in RET-positive patients, suggesting that vandetanib efficacy is independent of RET mutation status. In contrast, in the cabozantinib group, median PFS1 was not reached in RET-negative patients (80% remained on treatment at 25 months). In contrast, it decreased to 12.3 months in RET-positive patients, indicating reduced efficacy in the RET-mutant subgroup.

When evaluating other TKIs, first-line median PFS1 was 17 months with sorafenib, 9.6 months with sunitinib, and only 4.9 months with chemotherapy. Among alternative agents used when vandetanib or cabozantinib were unavailable or contraindicated, sorafenib showed numerically longer PFS1 compared with other alternatives; however, given the small patient numbers in each subgroup, this observation should be considered exploratory. In the second-line setting, median PFS2 was 17.8 months with sorafenib, 6.2 months with sunitinib, 6.1 months with lenvatinib, and 4.8 months with chemotherapy. Although inferior to vandetanib and cabozantinib, sorafenib appeared to be a reasonable option among other TKIs in this dataset. Chemotherapy showed limited efficacy in both first- and second-line settings.

The prognostic analyses from our cohort identified several clinically meaningful predictors of overall survival. Younger age (below 40 years) was independently associated with improved OS on multivariate analysis (HR 0.16, 95% CI 0.03–0.72; *p* = 0.017), consistent with prior observations that younger patients tend to harbor less aggressive disease biology and tolerate systemic therapy better. Female sex was associated with better OS in univariate analysis, though this association did not persist after multivariate adjustment, suggesting confounding by age or performance status. The presence of bone metastasis was independently associated with improved OS in the multivariate model (HR 0.29, 95% CI 0.11–0.73; *p* = 0.009). While this finding may appear counterintuitive, it could reflect treatment-related factors in this retrospective cohort, such as earlier initiation or more aggressive use of TKI therapy in patients with symptomatic skeletal involvement. This observation should be interpreted with caution, given the retrospective design and potential for selection bias, and warrants further investigation in prospective studies. These findings underscore the importance of considering patient age and metastatic pattern when counseling patients and selecting treatment intensity.

A potentially clinically relevant observation from this study is that the sequence of cabozantinib and vandetanib deployment may be associated with differences in time to second progression. Patients who received both agents sequentially across the first two lines of therapy achieved a median time to second progression of 114 months, compared with 39 months in patients who received at least one alternative TKI in either line (*p* = 0.003). However, given the retrospective design, small subgroup sizes, and potential for treatment-selection bias, these findings should be interpreted as exploratory and hypothesis-generating rather than as evidence of a causal relationship. The observed association suggests that preserving access to both approved aaMKIs may confer a cumulative benefit in disease control duration. The overall survival benefit associated with second-line cabozantinib or vandetanib use (HR 0.40, 95% CI 0.16–0.98; *p* = 0.046) provides further supportive, albeit hypothesis-generating, evidence for the clinical value of ensuring patients have access to both agents throughout their disease. The absence of a statistically significant OS difference between patients receiving sequential cabozantinib–vandetanib versus other TKI combinations (*p* = 0.071) may reflect inadequate statistical power due to sample size, the immaturity of OS data in a disease with naturally prolonged survival, and potential crossover effects in subsequent treatment lines.

RET mutation status did not emerge as a significant predictor of overall survival in our cohort, which may in part reflect the limited proportion of patients who underwent molecular testing (63.6%) and the small number with confirmed RET mutations (21.8%). In the present study, vandetanib appeared to retain efficacy irrespective of RET mutation status, while cabozantinib demonstrated numerically shorter PFS in RET-positive patients. These observations are consistent with prior subgroup analyses from the EXAM trial [20], which found that RET M918T mutation status influenced the magnitude of cabozantinib benefit; however, our findings should be interpreted cautiously given the small subgroup sizes. The underutilization of selective RET inhibitors, such as selpercatinib, in our cohort reflects the accessibility constraints prevalent in resource-limited healthcare settings and likely represents an unmet clinical need. Broader implementation of molecular testing and improved drug access will be essential for optimizing outcomes in RET-mutant disease.

The present study did not include a detailed safety and tolerability analysis. Due to the retrospective nature of the study and the variability in adverse event documentation across the 24 participating centers, a comprehensive toxicity grading analysis was not feasible. Nevertheless, it is important to acknowledge that both vandetanib and cabozantinib carry a substantial toxicity burden in clinical practice. Commonly reported adverse events include hypertension, diarrhea, palmar-plantar erythrodysesthesia, QTc prolongation, and hepatotoxicity, which frequently necessitate dose modifications, treatment interruptions, or discontinuation. These toxicity-related factors may have influenced treatment duration and, consequently, progression-free survival estimates in the present cohort. A systematic evaluation of treatment-related adverse events, dose reductions, and their impact on clinical outcomes represents an important area for investigation in future prospective real-world studies.

Several limitations of this study merit consideration. First, the retrospective design introduces inherent risks of selection bias and incomplete data capture, particularly regarding molecular profiling, biomarker assessment, and reasons for treatment discontinuation. Second, although this is one of the largest real-world multicenter MTC datasets reported from a single country, the absolute patient numbers in individual treatment subgroups remain modest, limiting the power of subgroup analyses and precluding definitive conclusions about rare treatment sequences. Third, drug supply interruptions affected a substantial proportion of patients receiving vandetanib (10.4%), potentially artificially attenuating PFS estimates in that group. Fourth, the relatively low rate of RET mutation testing reflects real-world practice constraints rather than scientific intent, and limits the generalizability of mutation-stratified analyses. Fifth, given the limited number of patients in specific sequencing subgroups, propensity score–adjusted analyses were not statistically feasible; consequently, the sequencing analyses remain susceptible to treatment-selection bias and should be interpreted as exploratory. Finally, overall survival data remain immature in several subgroups, and longer follow-up will be required before definitive conclusions about survival endpoints can be drawn. Despite these limitations, this study provides meaningful real-world evidence that complements and contextualizes data from registration trials conducted in predominantly Western patient populations.

## 5. Conclusions

Although RET mutation status is an important parameter in planning treatment for metastatic medullary thyroid carcinoma, challenges in accessing molecular testing and selective RET inhibitors (such as selpercatinib) may limit their routine use in developing countries. In such settings, vandetanib and cabozantinib demonstrate effective performance regardless of RET mutation status. Considering its efficacy in both the first-line setting and after progression on vandetanib, cabozantinib emerges as a particularly reliable agent. While guidelines recommend various TKIs after progression on these agents, real-world data from our study suggest that sorafenib may show numerically favorable outcomes compared with other alternative TKIs. However, given the small comparator group sizes, this observation should be regarded as exploratory and requires prospective validation before definitive clinical recommendations can be made.

## Figures and Tables

**Table 1:** General characteristics of the study population (*n* = 115). Continuous variables are expressed as median (range); categorical variables as *n* (%).

Variable	Category	*n* (%)
**Demographics**		
**Age, median (range), years**		47.4 (11–88)
	<40	33 (28.7%)
	40–65	69 (60.0%)
	>65	13 (11.3%)
**Sex**	Male	73 (63.5%)
	Female	42 (36.5%)
**ECOG Performance Status**	0–1	110 (95.7%)
	2–3	5 (4.3%)
**Family History of MTC**	None	98 (85.2%)
	Yes	17 (14.8%)
**Smoking Status**	Non-smoker	69 (60.0%)
	Active, <20 pack-years	9 (7.8%)
	Active, ≥20 pack-years	15 (13.0%)
**Comorbidities**		
**Any comorbidity**	None	79 (68.7%)
	Yes	36 (31.3%)
**Hypertension**		25 (21.7%)
**Diabetes mellitus**		14 (12.2%)
**Coronary artery disease**		12 (10.4%)
**TNM Classification**		
**T stage**	T1	20 (18.5%)
	T2	35 (32.4%)
	T3	36 (33.3%)
	T4	15 (13.9%)
**N stage**	N0	13 (11.8%)
	N1	94 (85.5%)
	N2	2 (1.7%)
	N3	1 (0.9%)
**Tumor laterality**	Unilateral	70 (64.8%)
	Bilateral	33 (30.6%)
	Unilateral multifocal	4 (3.7%)
**Surgical Treatment**		
**Type of thyroidectomy**	Total	87 (91.6%)
	Subtotal	6 (8.4%)
**Lymph node dissection**	Not performed	13 (13.3%)
	Unilateral	18 (18.4%)
	Bilateral	54 (55.1%)
	Central	13 (13.3%)
**Molecular Characteristics**		
**RET mutation status**	Negative	46 (40.0%)
	Positive	24 (20.9%)
	Not tested	40 (34.8%)
	Missing data	5 (4.3%)
**RAS mutation status**	Negative	37 (32.2%)
	Positive	1 (0.9%)
	Not tested	77 (67.0%)
**Metastatic Disease**		
**Metastasis at initial diagnosis**	No	60 (52.2%)
	Yes	55 (47.8%)
**Metastatic sites**	Lymph node	97 (84.3%)
	Bone	56 (48.7%)
	Lung	50 (43.5%)
	Liver	31 (27.0%)
	Other	10 (8.7%)
**Systemic Treatment**		
**First-line agent**	Vandetanib	55 (47.8%)
	Cabozantinib	35 (30.4%)
	Chemotherapy	15 (13.0%)
	Sorafenib	7 (6.1%)
	Other	3 (2.6%)
**Second-line agent**	Cabozantinib	26 (36.6%)
	Vandetanib	15 (21.1%)
	Sorafenib	15 (21.1%)
	Chemotherapy	8 (11.3%)
	Other	7 (9.8%)

Abbreviations: ECOG, Eastern Cooperative Oncology Group performance status; MTC, medullary thyroid carcinoma; RET, rearranged during transfection; RAS, rat sarcoma viral proto-oncogene.

**Table 2:** Cox regression analysis of variables according to OS.

Variables	Overall Survival			
	Univariate HR (95% CI)	*p*	Multivariate HR (95% CI)	*p*
**Age (<40 vs. ≥40)**	0.19 (0.06–0.65)	0.008	0.16 (0.03–0.719)	0.017
**Gender (Female vs. Male)**	0.40 (0.16–0.98)	0.047	0.42 (0.12–1.46)	0.174
**Family History (no vs. yes)**	1.71 (0.518–5.64)	0.379		
**Smoke (no vs. yes)**	0.66 (0.31–1.37)	0.266		
**Alcohol (no vs. yes)**	0.55 (0.16–1.83)	0.33		
**Comorbidity (without vs. with)**	0.53 (0.26–1.09)	0.087		
**Calcitonin (<1000 vs. ≥1000)**	0.47 (0.18–1.17)	0.10		
**CEA (low vs. high)**	0.26 (0.03–1.96)	0.19		
**RET mutation (negative vs. positive)**	1.06 (0.27–4.11)	0.92		
**Lymph node metastasis (no vs. yes)**	1.70 (0.76–3.82)	0.19		
**Lung metastasis (no vs. yes)**	1.74 (0.83–3.63)	0.14		
**Bone metastasis (yes vs. no)**	0.48 (0.23–0.98)	0.046	0.29 (0.11–0.73)	0.009
**Liver metastasis (no vs. yes)**	0.78 (0.36–1.67)	0.53		
**1L Cabozantinib or Vandetanib (yes vs. no)**	0.77 (0.36–1.64)	0.50		
**2L Cabozantinib or Vandetanib (yes vs. no)**	0.38 (0.15–0.92)	0.032	0.40 (0.163–0.983)	0.046

## Data Availability

The original contributions presented in this study are included in the article. Further inquiries can be directed to the corresponding author.
